# A de novo mutation in *KRT5* in a crossbred calf with epidermolysis bullosa simplex

**DOI:** 10.1111/jvim.15943

**Published:** 2020-11-02

**Authors:** Joana G. P. Jacinto, Irene M. Häfliger, Inês M. B. Veiga, Cord Drögemüller, Jørgen S. Agerholm

**Affiliations:** ^1^ Department of Veterinary Medical Sciences University of Bologna Bologna Italy; ^2^ Institute of Genetics, Vetsuisse Faculty, University of Bern Bern Switzerland; ^3^ Institute of Animal Pathology, Vetsuisse Faculty, University of Bern Bern Switzerland; ^4^ Department of Veterinary Clinical Sciences University of Copenhagen Copenhagen Denmark

**Keywords:** cattle, KRT5, precision medicine, skin fragility, WGS

## Abstract

A 6‐day‐old Belgian Blue‐Holstein calf was referred because of a syndrome resembling epidermolysis bullosa simplex (EBS). The clinical phenotype included irregular and differently sized erosions and ulcerations spread over the body, in particular on the limbs and over bone prominences, as well as in the nasal planum and oral mucosa. Blisters were easily induced by rubbing the skin. The skin lesions displayed a clear dermal‐epidermal separation at the level of the basal cell layer. Post mortem examination revealed erosions in the pharynx, proximal esophagus, and rumen. Whole‐genome sequencing revealed a heterozygous disruptive in‐frame deletion variant in *KRT5* (c.534_536delCAA). Genotyping of both parents confirmed the variant as de novo mutation. Clinicopathological and genetic findings were consistent with the diagnosis of *KRT5*‐related EBS providing the second example of a spontaneous mutation causing epidermolysis bullosa in cattle.

**Abbreviations**
DEB: dystrophic EB
EBS: EB simplex
EDTA: ethylenediaminetetraacetic acid
EB: epidermolysis bullosa
HE: hematoxylin and eosin
HIM: helix initiation peptide motif
HTM: helix termination peptide motif
IGV: Integrative Genomics Viewer
JEB: junctional EB
KRT5: keratin 5
KEB: Kindler EB
WGS: whole‐genome sequencing

## 1 INTRODUCTION

Epidermolysis bullosa (EB) encompasses a heterogeneous group of genetic mechanobullous disorders characterized by blistering from even minor mechanical trauma with disruption at the dermal‐epidermal junction.^1^ Epidermolysis bullosa disorders are characterized by clinical heterogeneity, both in their appearance and severity. The disease might be congenital or develop later in life. In congenital cases, the lesions are more severe, accompanied by mucosal fragility, and might involve other organs than the skin.^2^ In noncongenital cases, the skin fragility is less severe and the lesions are usually localized to the extremities of the limbs, occasionally only expressed as nail/hoof dystrophy.^3^ Based on the ultrastructural level of skin cleavage, there are 4 major classical types: EB simplex (EBS), junctional EB (JEB), dystrophic EB (DEB), and Kindler EB (KEB).^4^ In EBS, the skin cleavage occurs within the epidermis, in JEB within the lamina lucida and in DEB within the superficial dermis. Kindler EB might present with all 3 cleavage levels.^3^ The same classification might be used in veterinary medicine; however, cases of KEB are not reported in domestic animals. In human, these phenotypical classifications are complicated by the fact that, depending on the variant, the same gene might be associated with different modes of inheritance, thus resulting in distinct clinical phenotypes.^5^ Dystrophic EB and EBS phenotypes might be inherited either dominantly or recessively, and might be caused by pathogenic variants in different genes.^4^ In human medicine, EB is associated with more than 1000 variants in at least 18 genes encoding structural proteins.^1^, ^5^

Four EB‐related causative recessive variants are known in cattle,^6^, ^7^, ^8^, ^9^ 3 in dogs,^10^, ^11^, ^12^, ^13^ 2 in sheep,^14^, ^15^ and 2 in horses,^16^, ^17^, ^18^, ^19^ and 1 dominant variant is known in cattle^20^ (Table S1). For cattle, a dominant form of EBS is associated with a keratin 5 (*KRT5*) missense variant (OMIA 002081‐9913),^20^ and recessive forms of JEB are associated with deleterious variants in *ITGB4* (OMIA 001948‐9913), *LAMA3* (OMIA 001677‐9913), and *LAMC2* (OMIA 001678‐9913).^6^, ^7^, ^8^ In addition, a form of recessive DEB is associated with a nonsense variant in *COL7A1* (OMIA 000341‐991).^9^

Older reports of familial occurrence of EB,^21^ outbreaks of several inherited‐related cases in single herds^21^, ^22^, ^23^ and sporadic cases of EB^24^, ^25^, ^26^, ^27^ in cattle exist. In the abovementioned cases,^21^, ^22^, ^23^ the diagnosis was based only on the clinical and histopathological findings.

The majority of the previous reports focused on disorders with a recessive inheritance. However, single cases because of dominant acting de novo variants might occur sporadically without impact on breeding. At present, this obvious genetic heterogeneity could be analyzed in cattle using whole‐genome sequencing (WGS)‐based precision diagnostics.^28^ Therefore, the purpose of this study was to characterize the clinical and pathological phenotype of an EBS‐affected calf, and to evaluate its possible genetic etiology using WGS.

## 2 CASE DESCRIPTION

A 6‐day‐old (46 kg) male Belgian Blue‐Holstein crossbred calf was submitted for clinical investigation because of ulcerations of the skin and nasal planum since short time after birth. The animal was delivered after a gestation period of 287 days.

**FIGURE 1:**
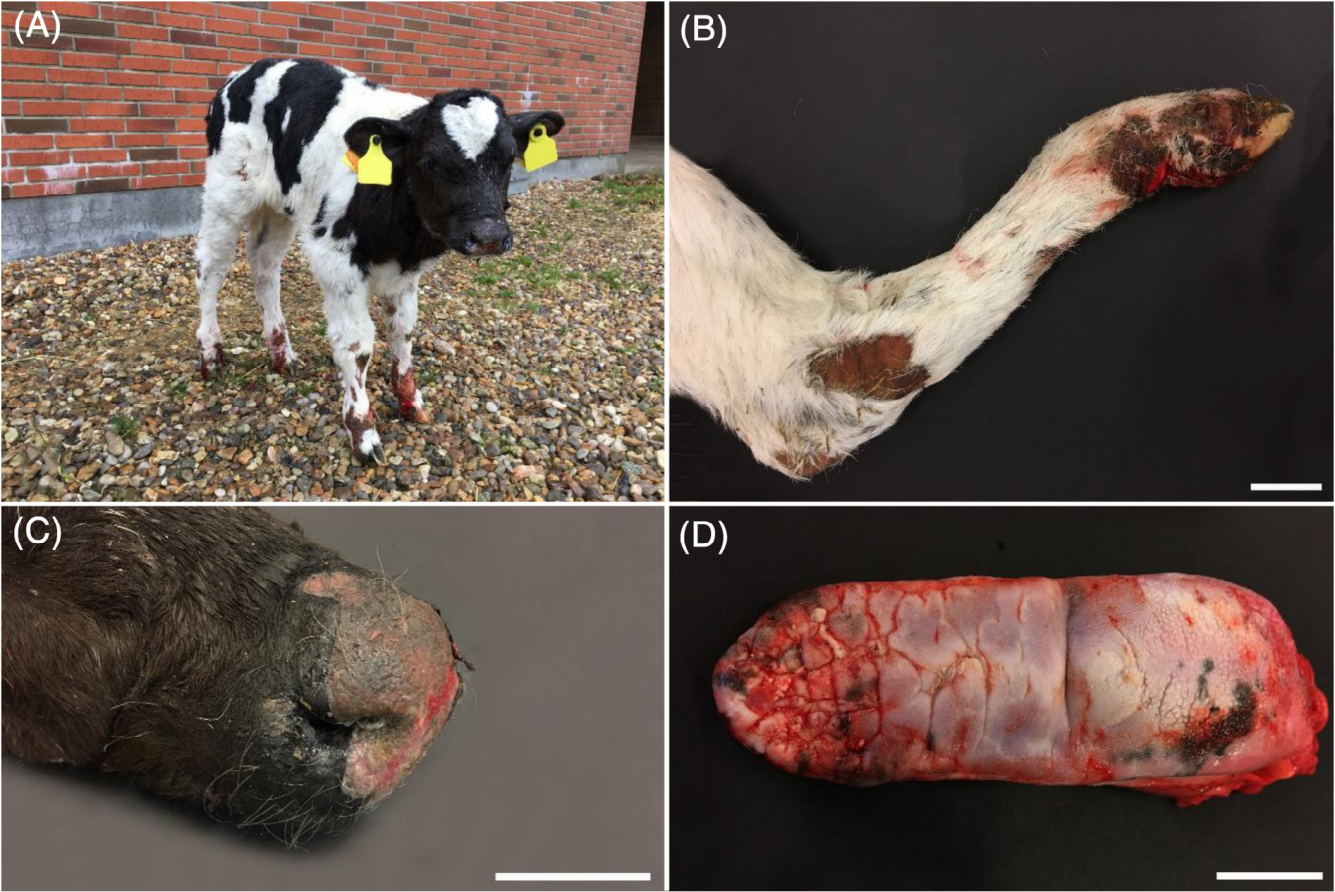
Lesions in the affected calf. A, Widespread irregular ulcerations of various sizes on most parts of the body. B, Irregular ulceration on the hindlimb. Scale bar = 5 cm. C, Extensive ulcerations on the nasal planum, lips and nares; also, purulent nasal discharge was present. Scale bar = 2.5 cm. D, Thickened and the furrows of the epithelium on the dorsal surface of the tongue. Scale bar = 5 cm

The cutaneous lesions were characterized by widespread irregular erosions and ulcerations of various sizes on most parts of the body (Figure 1A), but in particular on the limbs (Figure 1B) and over bony prominences. Upon handling, the epidermis easily separated leaving a blister with a black colored, nonhemorrhagic base indicating a separation superficial to the stratum basale. Peracute blister were easily induced by rubbing the skin by an eraser after having cut the hair locally. Older lesions consisted of ulcerations covered by crusts and occasional acute hemorrhage. On the nasal platum, lips and nares extensive ulcerations were present; the calf also showed a purulent nasal discharge (Figure 1C). Moreover, the animal seemed to be in pain when walking on a hard surface. The aspect resembled EB and therefore was further referred to the Danish surveillance program for genetic diseases in cattle for further examination. Because of the poor prognosis and the painful situation, the calf was euthanized for welfare reasons by IV administration of an overdose of pentobarbital. In addition to the skin lesions, gross pathologic examination revealed erosions in the oral cavity, pharynx, proximal esophagus, and rumen. The epithelium on the dorsal surface of the tongue was thickened and with furrows (Figure 1D). The incisor teeth were disorganized and not completely erupted and the surrounding parts of the mandibles appeared thickened and cystic. The hoofs seemed intact, yet when sawed longitudinally, the capsule was partly separated from the dermal lamella with hemorrhage in the interface.

**FIGURE 2:**
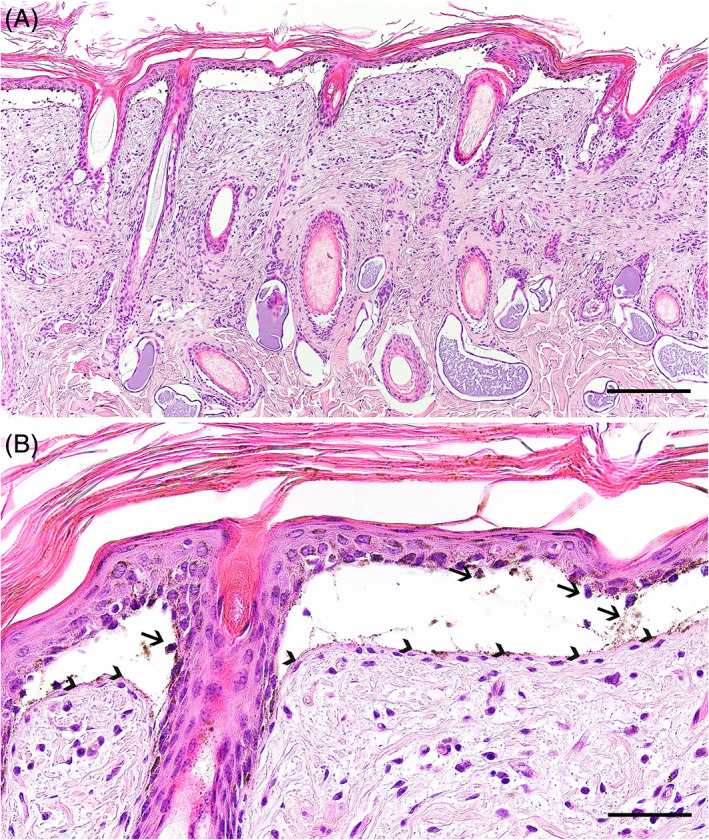
Histopathological findings of the affected calf. A, The peracute cutaneous lesions displayed an extensive dermal‐epidermal separation at the level of the basal layer, which also affected the wall of the hair follicle infundibula. HE staining, scale bar = 200 μm. B, Higher magnification of the dermal‐epidermal detachment at the basal cell layer level, where it is possible to observe the presence of occasional basal cell remains within the cleft (arrows) and the basement membrane overlying the dermis (arrowheads). HE staining, scale bar = 50 μm

Immediately before euthanasia, the skin covering the dorsal part of the pelvis was gently scrubbed with an eraser with blister formation. Skin biopsies from this area and from other representative cutaneous lesions were taken immediately after euthanasia for histological analysis, whereas the necropsy was completed at the university a few hours later. Additional specimens for histological analysis were then collected, including the oral mucosa, pharynx, rumen, reticulum, and major internal organs. All collected samples were fixed in 10% neutral buffered formalin, trimmed, processed, embedded in paraffin wax, sectioned at 4 to 5 μm, and stained by hematoxylin and eosin (HE). Histologically, the peracute lesions induced by rubbing before euthanasia displayed a very striking, multifocal to coalescing dermal‐epidermal separation at the level of the basal layer, which extended into the wall of the hair follicle infundibula (Figure 2). The spontaneously occurring, chronic lesions present in the nasal planum and in the distal limbs displayed a multifocal to coalescing epithelial loss with consequent severe ulceration and underlying neutrophilic infiltration, replacement of the papillary dermis by granulation tissue, and re‐epithelialization. A multifocal dermal‐epidermal separation at the level of the basal cell layer with multifocal underlying accumulation of free erythrocytes and fibrin exudation was occasionally visible at the border of the ulcerated areas.

In the tongue, the mucosa of the dorsal surface showed a marked parakeratotic hyperkeratosis. At the lateral borders, where the epithelium had a normal thickness, areas with complete loss of mucosa were observed. The superficial layer of the submucosal connective tissue had a necrotic surface, intense hyperemia, and infiltration with neutrophils. Mucosa cleavage in the adjacent areas was not observed, but the height of the epithelium gradually decreased. The stratum spinosum showed ballooning degeneration and in these areas, the stratum corneum was not present. In the pharyngeal lining, multiple intensely inflamed ulcers covered by a debris of fibrin, degenerated neutrophils, erythrocytes, bacterial colonies were present.

The epithelium of the rumen and reticulum was normally developed but an acute suppurative multifocal rumenitis was present. Separation of the epithelium from the underlying submucosa was seen in some areas but considered as a post mortem artifact. Additional findings included suppurative periportal hepatitis and multifocal intense pulmonary hyperemia associated with fibrin in the alveoli. Other tissues were unremarkable. The histopathological findings in the skin and pharyngeal linings resembled EBS.

Additionally, WGS using the Illumina NovaSeq6000 was performed on DNA extracted from ethylenediaminetetraacetic acid (EDTA) blood of the calf. The sequenced reads were mapped to the ARS‐UCD1.2 reference genome resulting in an average read depth of approximately 19×,^29^ and single‐nucleotide variants and small indel variants were called. The applied software and steps to process fastq files into binary alignment map and genomic variant call format files were in accordance with the 1000 Bull Genomes Project processing guidelines of run 7 (www.1000bullgenomes.com),^30^ except for the trimming, which was performed using fastp.^31^ Further preparation of the genomic data had been done according to Häfliger et al.^32^ In order to find private variants, we compared the genotypes of the affected calf with 493 cattle genomes of various breeds that had been sequenced in the course of other ongoing studies and that are publicly available (Table S2) in the European Nucleotide Archive (SAMEA6528898 is the sample accession number of the case; http://www.ebi.ac.uk/en). Integrative Genomics Viewer (IGV)^33^ software was used for visual inspection of candidate variants. A total of 115 private protein‐changing variants with a moderate or high predicted impact on the encoded protein, located within 108 different genes or loci, were identified. These variants were further checked for their occurrence in a global control cohort of 3103 genomes of a variety of breeds (1000 Bull Genomes Project run 7; www.1000bullgenomes.com), which revealed 26 protein‐changing variants exclusively present heterozygous in the genome of the EBS‐affected calf. These 26 variants located within 25 different genes or loci (Table S3) were subsequently visually inspected using IGV software confirming all as true variants. Of all these 26 remaining private variants, only 1 occurred in a candidate for EB: keratin 5 (*KRT5*). The variant was a heterozygous disruptive in‐frame deletion variant on chromosome 5: 27367604delCAA (NM_001008663.1:c.534_536delCAA), leading to a loss of an asparagine amino acid at residue 178 of the encoded *KRT5* protein (NP_001008663.1:p.Asn178del). This variant affecting an EB candidate gene was further investigated as likely causal mutation for the observed phenotype.

**FIGURE 3:**
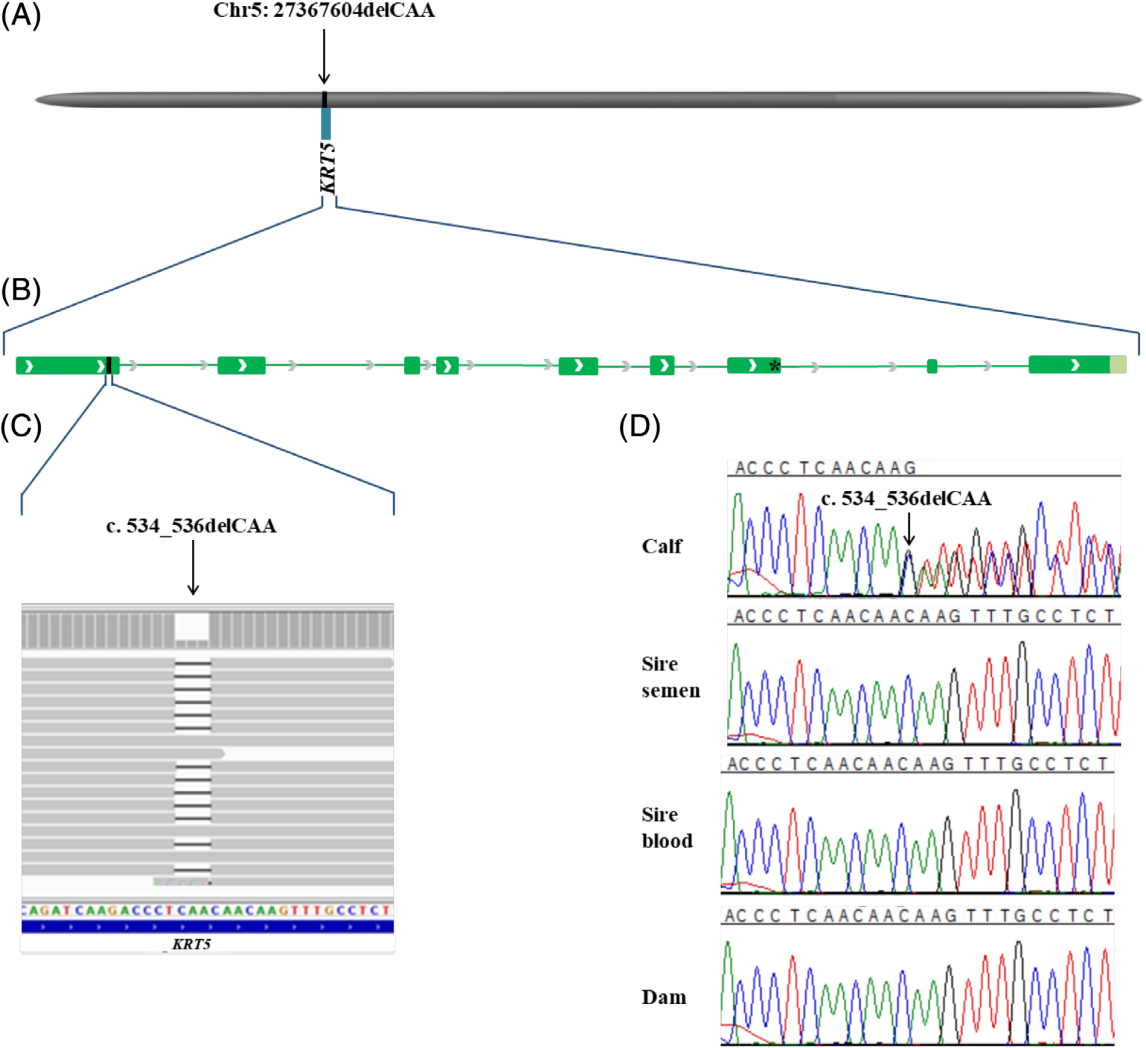
Schematic diagram of the *KRT5* gene showing the location of the candidate causal variant NM_001008663.1:c.534_536delCAA of the affected calf. A, Location of the bovine *KRT5* gene, Chr5:27 367 078‐27 372 929 and causal variant, Chr5:27 367 604 on the ARS‐UCD1.2 bovine genome assembly. B, Genomic structure of *KRT5* gene. Green boxes represent the exons. The c.534_536delCAA is located in the 1st exon of KRT5 gene. The previous reported g.27371128G>A missense variant is located in the 7th exon of *KRT5* gene. C, Integrative Genomics Viewer (IGV) screenshot presenting the *KRT5* variant. D, Sanger sequencing results confirmed that the variant occurred de novo as sequencing of PCR products from DNA of both parents (for the sire both semen and blood) showed that the variant was absent

To confirm that the c.534_536delCAA variant in *KRT5* was a de novo mutation, the affected genomic region was amplified by polymerase chain reaction (PCR) and Sanger sequenced in the affected calf, its Belgian Blue sire and Holstein dam based on DNA extracted from EDTA blood of the dam, and from both EDTA blood and semen of the sire. PCR products were amplified using flanking primers for the *KRT5* exon 1 deletion with 5′‐AGGCATCCAAGAGGTCACCG‐3′ (forward primer) and 5′‐TAGCACATATCCCACACTCATGG‐3′ (reverse primer). Sequence data were analyzed using Sequencher 5.1 (GeneCodes). Analyzing the sequencing data, we concluded that only the EBS‐affected calf was heterozygous for the *KRT5* variant and the dam and sire were both homozygous for the wild type allele in all analyzed samples including both semen and blood of the sire (Figure 3).

## 3 DISCUSSION

The clinical and pathological findings in the calf were consistent with EB. Although most lesions had the appearance of unspecific inflamed ulcerations, which usually develop shortly after dermal‐epidermal separation, blistering could be easily induced in intact skin by rubbing the skin surface. Histopathological analysis of these lesions showed that the dermal‐epidermal separation occurred at the level of the basal cell layer, which is suggestive for EBS.

**FIGURE 4:**
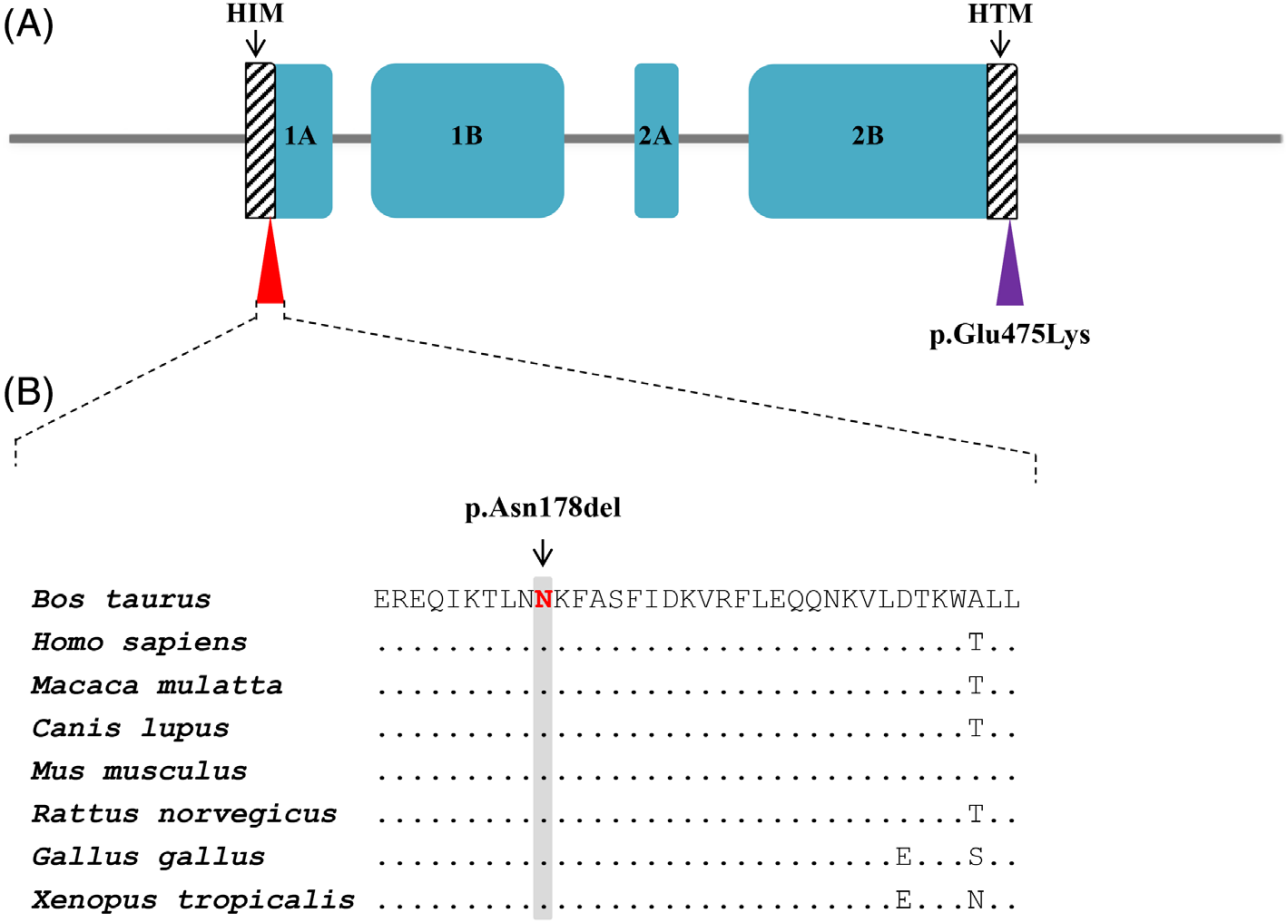
Schematic diagram of the KRT5 protein. A, Domain and region information of KRT5 protein obtained from the UniProt database (http://www.uniprot.org/; accession number: Q5XQN5). The p.Asn178del variant in the helix initiation peptide motif (HIM) is indicated by the red triangle. The EBS‐causing p.Glu475Lys variant previously reported^20^ in the helix termination peptide motif (HTM) is indicated by a violet triangle. The arrows indicate the HIM and the HTM. B, Multiple sequence alignment of 1A rod domain of the of KRT5 protein encompassing the region of the p.Asn178del variant demonstrates a complete evolutionary conservation across species. The observed variant is indicated by an arrow and the respective position highlighted in gray. Protein sequences accession numbers in NCBI for each species are NP_001008663.1 (*Bos taurus*), NP_000415.2 (*Homo sapiens*), XP_002798641.1 (*Macaca mulatta*), XP_005636850.1 (*Canis familiaris*), NP_081287.1 (*Mus musculus*), NP_899162.1 (*Rattus norvegicus*), NP_001001195.1 (*Gallus gallus*), and NP_001072377.1 (*Xenopus tropicalis*)

The predicted deleterious protein effect c.534_536delCAA variant and the conservation of the affected asparagine amino acid residue at position 178 in the helix initiation motif (HIM) of the highly conserved 1A rod domain of *KRT5* suggest that this de novo mutation variant is certainly pathogenic (Figure 4). The mutation most likely occurred post‐zygotically during the calf's fetal development as it was absent in both parents. In cattle, a de novo missense variant in *KRT5* is reported in an asymptomatic Friesian‐Jersey crossbred mosaic sire and EBS‐affected offspring. That mutation results in an amino acid exchange (p.Glu478Lys) in the final glutamic acid of the KLLEGE motif of the highly conserved 2B rod domain of *KRT5* (Figure 4).^20^

The early onset combined with multifocal to widespread lesions in the skin and mucosal membranes classifies this condition as a severe form of EB. The reported bovine *KRT5* associated EB case also had such lesions^20^; this indicates that mutations in the *KRT5* in cattle might cause severe EBS when present. Histologically the present case showed suppurative rumenitis and rumen epithelial detachment. Because of the delayed necropsy, the latter might have been a post mortem artifact. However, no ulcerations were observed in the forestomach and inflamed areas were covered by an epithelium, therefore a possible association with EB remains hypothetical.

The case of EB presented in this study can be classified as EBS. In humans, among the several subtypes reported in the literature, the most common EBS subtypes might be considered the so‐called localized, severe, and intermediate form. In the localized EBS, the blisters are present only on the extremities of the limbs. Rare phenotypical subtypes of localized EBS are associated with nephropathy.^34^ In severe forms of EBS the lesions are present from birth, are more diffuse on the body, with main severity on the extremities and over bone prominences, and deep ulceration might be observed. After some time, large tense blisters might arise spontaneously or secondary to minor trauma. The blisters characteristically have an arciform pattern and eventuate with crusts to necrosis with a visual similarity to inflammatory plaques.^1^ Also, the oral mucosa might be affected. Some phenotypical subtypes of the severe EBS are accompanied by other systemic complications with subsequent growth retardation, nutritional deficiency or even lethal outcome because of secondary infections or respiratory failure.^35^ In intermediate EBS the skin lesions are diffuse on the body, however they are not so serious as in the severe EBS. Rare phenotypical subtypes of intermediate EBS are accompanied by cardiomyopathy^36^, ^37^ and muscular dystrophy.^38^, ^39^

In human, intermediate EBS with muscular dystrophy is often associated with enamel hypoplasia.^39^ Furthermore, beyond the characteristic skin lesions, it might include diffuse alopecia, short stature, slow weight gain, punctate keratitis, urethral strictures, muscular dystrophy, and degenerative changes with increased connective tissue.^35^ Such findings were not observed in the studied calf.

In human medicine, a de novo missense variant in *KRT5* resulting in an amino acid exchange (p.Asn177Ser) in the HIM has been reported in a patient with localized EBS.^40^ This human KRT5 protein position corresponds to the position of the p.Asn178del variant present in this case. In human medicine, the localized, severe, and intermediate subtypes of the EBS are mostly linked to an autosomal dominant pattern and are associated to a high rate of de novo mutations. The mutation in the present case was in that aspect similar to the human cases, in that it was autosomal dominant and of de novo origin. The most common mutations are caused by monoallelic pathogenic missense, nonsense, frameshift, or splice site variants or in frame deletions in *KRT5* and *KRT14*.^41^ However, rare EBS subtypes might be associated with pathogenic variants in other genes, such as *EXPH5*,^42^ *KLHL24*,^43^ *DST*,^44^ *PLEC*,^45^, ^46^, ^47^, ^48^ and *CD151*.^34^

Epidermolysis bullosa simplex is a rare disorder known in man and animals. Rare disorders such as EBS in livestock are usually not diagnosed to the molecular level, mainly because of lack of resources and diagnostic tools as well as low value and often‐short lifespan of the animals. The report of this case allowed the performance of a complete clinical, pathological, and molecular genetic study enabling the diagnosis of a severe form of EBS. Furthermore, this example highlights the utility of WGS‐based precision diagnostics for understanding rare disorders in animals with an available reference genome sequence and the value of surveillance of cattle breeding populations for harmful genetic disorders.

## CONFLICT OF INTEREST DECLARATION

Authors declare no conflicts of interests.

## OFF‐LABEL ANTIMICROBIAL DECLARATION

Authors declare no off‐label use of abtimicrobials.

## INSTITUTIONAL ANIMAL CARE AND USE COMMITTEE (IACUC)

This study was not based on an invasive animal experiment but was based on a spontaneously occurring case; therefore, there are no associated permit numbers.

## HUMAN ETHICS APPROVAL DECLARATION

Authors declare human ethics approval was not needed for this study.

## Supporting information


**Table S1** Classification of classical EB and correspondent known causative genetic variants in domestic animals.

**Table S2** EBI Accession numbers of all publicly available genome sequences. We compared the genotypes of the calf with 493 cattle genomes of various breeds that had been sequenced in the course of other ongoing studies and that were publicly available.

**Table S3** List of the remaining variants after the comparison to the global control cohort of 3103 genomes of other breeds (1000 Bull Genomes Project run 7; www.1000bullgenomes.com), revealing 26 protein‐changing variants only present in the genome of the EBS‐affected calf. These 26 variants with a moderate or high predicted impact on the encoded protein were located within 25 different genes or loci. Note that the predicted pathogenic variant NM_001008663.1:c.534_536delCAA is the only 1 located in a candidate gene.
