# Supplementary material for: A de novo mutation in KRT5 in a crossbred calf with epidermolysis bullosa simplex
Source: J Vet Intern Med. 2020 Nov 2;34(6):2800–7. doi: 10.1111/jvim.15943 (PMC7694802; doi:10.1111/jvim.15943)
Supplement: Supplementary file 1 — Table S1 Classification of classical EB and correspondent known causative genetic variants in domestic animals. [file JVIM-34-2800-s001.pdf]

Supplementary Table 1. Classification of classical EB and correspondent known causative genetic variants in domestic animals.

Classical types of EB level of skin cleavage	EB type	Inheritance	Affected gene(s)	Targeted protein(s)	Type of variant	Species	Breed(s)	OMIA*	References
Intraepidermal	EBS	AD	*KRT5*	Keratin 5	Missense	*Bos taurus*	Friesian-Jersey	OMIA 002081-9913	19
		AR	*PLEC*	Plectin	Nonsense (stop-gain)	*Canis lupus familiaris*	Eurasier	OMIA 002080-9615	10
Junctional	JEB	AR	*LAMA3*	Laminin subunit alpha 3	Nonsense (stop-gain)	*Bos taurus*	Belgian Blue	OMIA 001677-9913	7
					deletion, gross (>20)	*Equus caballus*	American Saddlebred	OMIA 001677-9796	16
					insertion, gross (>20)	*Canis lupus familiaris*	German pointer	OMIA 001677-9615	11
			LAMC2	Laminin subunit gamma 2	Deletion, gross (> 20)	*Bos taurus*	Hereford	OMIA 001678-9913	8
					Deletion, small (<=20)	*Ovis aries*	German Blackheaded Mutton	OMIA 001678-9940	15
					Insertion, small (<=20)	*Equus caballus*	Belgian; Italian draft horse; Trait Breton; Trait Comtois	OMIA 001678-9796	12, 18, 19
			ITGB4	Integrin subunit beta 4	Deletion, gross (> 20)	*Bos taurus*	Charolais	OMIA 001948-9913	6
					deletion, small (<=20)	*Ovis aries*	Churra	OMIA 001948-9940	14
Dermal	DEB	AR	*COL7A1*	Collagen type VII alpha 1 chain	Nonsense (stop-gain)	*Bos taurus*	Rotes Höhenvieh, Vorderwald	OMIA 000341-9913	9
						Canis lupus familiaris	Central Asian Shepherd	OMIA 000341-9615	12
					Missense	*Canis lupus familiaris*	Golden Retriever	OMIA 000341-9615	13

AD, autosomal dominant; AR, autosomal recessive. *Online Mendelian Inheritance in Animals, http://omia.angis.org.au/.
