# Supplementary material for: A de novo mutation in KRT5 in a crossbred calf with epidermolysis bullosa simplex
Source: J Vet Intern Med. 2020 Nov 2;34(6):2800–7. doi: 10.1111/jvim.15943 (PMC7694802; doi:10.1111/jvim.15943)
Supplement: Supplementary file 2 — Table S2 EBI Accession numbers of all publicly available genome sequences. We compared the genotypes of the calf with 493 cattle genomes of various breeds that had been sequenced in the course of other ongoing studies and that were publicly available. [file JVIM-34-2800-s002.pdf]

Table S2: EBI Accession numbers of all publicly available genome sequences.

EBI Project ID	EBI Sample ID
PRJEB18113	SAMEA5159765
PRJEB18113	SAMEA5159766
PRJEB18113	SAMEA5159767
PRJEB18113	SAMEA5159768
PRJEB18113	SAMEA5159769
PRJEB18113	SAMEA5159770
PRJEB18113	SAMEA5159771
PRJEB18113	SAMEA5159772
PRJEB18113	SAMEA5159773
PRJEB18113	SAMEA5159774
PRJEB18113	SAMEA5159775
PRJEB18113	SAMEA5159776
PRJEB18113	SAMEA5159777
PRJEB18113	SAMEA5159778
PRJEB18113	SAMEA5159779
PRJEB18113	SAMEA5159780
PRJEB18113	SAMEA5159781
PRJEB18113	SAMEA5159782
PRJEB18113	SAMEA5159783
PRJEB18113	SAMEA5159784
PRJEB18113	SAMEA5159785
PRJEB18113	SAMEA5159786
PRJEB18113	SAMEA5159787
PRJEB18113	SAMEA5159788
PRJEB18113	SAMEA5159789
PRJEB18113	SAMEA5159790
PRJEB18113	SAMEA5159791
PRJEB18113	SAMEA5159792
PRJEB18113	SAMEA5159793
PRJEB18113	SAMEA5159794
PRJEB18113	SAMEA5159795
PRJEB18113	SAMEA5159796
PRJEB18113	SAMEA5159797
PRJEB18113	SAMEA5159798
PRJEB18113	SAMEA5159799
PRJEB18113	SAMEA5159800
PRJEB18113	SAMEA5159801
PRJEB18113	SAMEA5159802
PRJEB18113	SAMEA5159803
PRJEB18113	SAMEA5159804

PRJEB18113	SAMEA5159805
PRJEB18113	SAMEA5159806
PRJEB18113	SAMEA5159807
PRJEB18113	SAMEA5159808
PRJEB18113	SAMEA5159809
PRJEB18113	SAMEA5159810
PRJEB18113	SAMEA5159811
PRJEB18113	SAMEA5159812
PRJEB18113	SAMEA5159813
PRJEB18113	SAMEA5159814
PRJEB18113	SAMEA5159815
PRJEB18113	SAMEA5159816
PRJEB18113	SAMEA5159817
PRJEB18113	SAMEA5159818
PRJEB18113	SAMEA5159819
PRJEB18113	SAMEA5159820
PRJEB18113	SAMEA5159821
PRJEB18113	SAMEA5159822
PRJEB18113	SAMEA5159823
PRJEB18113	SAMEA5159824
PRJEB18113	SAMEA5159825
PRJEB18113	SAMEA5159826
PRJEB18113	SAMEA5159827
PRJEB18113	SAMEA5159828
PRJEB18113	SAMEA5159760
PRJEB18113	SAMEA5159761
PRJEB12093	SAMEA3706825
PRJEB18113	SAMEA5415491
PRJEB14604	SAMEA4051550
PRJEB18113	SAMEA4644752
PRJEB18113	SAMEA4644757
PRJEB18113	SAMEA4644727
PRJEB18113	SAMEA4644734
PRJEB18113	SAMEA5714976
PRJEB18113	SAMEA19315168
PRJEB18113	SAMEA5415485
PRJEB18113	SAMEA6528890
PRJEB18113	SAMEA4644764
PRJEB18113	SAMEA5714974
PRJEB18113	SAMEA5159847
PRJEB18113	SAMEA5159886
PRJEB18113	SAMEA4644728
PRJEB18113	SAMEA4644749

PRJEB18113	SAMEA6528886
PRJEB18113	SAMEA4644750
PRJEB18113	SAMEA6528894
PRJEB18113	SAMEA6528892
PRJEB18113	SAMEA4644762
PRJEB18113	SAMEA4644739
PRJEB18113	SAMEA4644755
PRJEB18113	SAMEA5415486
PRJEB18113	SAMEA5564716
PRJEB18113	SAMEA5564728
PRJEB18113	SAMEA19312918
PRJEB18113	SAMEA5415489
PRJEB18113	SAMEA4644763
PRJEB18113	SAMEA4644766
PRJEB18113	SAMEA19323418
PRJEB18113	SAMEA5714979
PRJEB18113	SAMEA6528889
PRJEB18113	SAMEA19313668
PRJEB18113	SAMEA4644769
PRJEB18113	SAMEA19314418
PRJEB18113	SAMEA4644754
PRJEB18113	SAMEA5159853
PRJEB18113	SAMEA4644765
PRJEB18113	SAMEA4644768
PRJEB18113	SAMEA5415488
PRJEB18113	SAMEA6528891
PRJEB18113	SAMEA5714971
PRJEB18113	SAMEA6528888
PRJEB18113	SAMEA4644741
PRJEB28191	SAMEA4827664
PRJEB28191	SAMEA4827655
PRJEB18113	SAMEA4644743
PRJEB28191	SAMEA4827671
PRJEB28191	SAMEA4827649
PRJEB18113	SAMEA5159875
PRJEB28191	SAMEA4827653
PRJEB28191	SAMEA4827657
PRJEB28191	SAMEA4827663
PRJEB28191	SAMEA4827661
PRJEB28191	SAMEA4827662
PRJEB28191	SAMEA4827658
PRJEB28191	SAMEA4827646
PRJEB28191	SAMEA4827659

PRJEB28191	SAMEA4827652
PRJEB28191	SAMEA4827656
PRJEB28191	SAMEA4827669
PRJEB28191	SAMEA4827651
PRJEB28191	SAMEA4827645
PRJEB28191	SAMEA4827660
PRJEB28191	SAMEA4827650
PRJEB18113	SAMEA4644758
PRJEB28191	SAMEA4827666
PRJEB18113	SAMEA5159861
PRJEB18113	SAMEA5159862
PRJEB28191	SAMEA4827647
PRJEB28191	SAMEA4827674
PRJEB28191	SAMEA4827667
PRJEB28191	SAMEA5059742
PRJEB28191	SAMEA4827654
PRJEB28191	SAMEA4827665
PRJEB28191	SAMEA4827670
PRJEB18113	SAMEA4644740
PRJEB18113	SAMEA4644742
PRJEB28191	SAMEA4827648
PRJEB28191	SAMEA4827668
PRJEB28191	SAMEA4827673
PRJEB18113	SAMEA5159849
PRJEB28191	SAMEA4827672
PRJEB18113	SAMEA5159867
PRJEB28191	SAMEA5059748
PRJEB18113	SAMEA19318918
PRJEB18113	SAMEA5159869
PRJEB18113	SAMEA5159870
PRJEB28191	SAMEA5059741
PRJEB18113	SAMEA5415498
PRJEB28191	SAMEA5059750
PRJEB28191	SAMEA5059751
PRJEB28191	SAMEA5059747
PRJEB28191	SAMEA5059759
PRJEB18113	SAMEA5159850
PRJEB28191	SAMEA5059743
PRJEB28191	SAMEA5059754
PRJEB28191	SAMEA5059749
PRJEB18113	SAMEA5159872
PRJEB28191	SAMEA5059758
PRJEB28191	SAMEA5059752

PRJEB28191	SAMEA5059745
PRJEB18113	SAMEA5159885
PRJEB28191	SAMEA5059757
PRJEB28191	SAMEA5059746
PRJEB18113	SAMEA5159873
PRJEB28191	SAMEA5059756
PRJEB28191	SAMEA5059744
PRJEB28191	SAMEA5059755
PRJEB18113	SAMEA4644756
PRJEB18113	SAMEA5159871
PRJEB28191	SAMEA5059753
PRJEB18113	SAMEA5159848
PRJEB18113	SAMEA6528893
PRJEB18113	SAMEA5159868
PRJEB18113	SAMEA4644730
PRJEB18113	SAMEA5159837
PRJEB18113	SAMEA5159843
PRJEB18113	SAMEA5714972
PRJEB18113	SAMEA6528895
PRJEB18113	SAMEA5714975
PRJEB18113	SAMEA6528887
PRJEB18113	SAMEA5564726
PRJEB18113	SAMEA5564727
PRJEB18113	SAMEA5415490
PRJEB18113	SAMEA5159874
PRJEB18113	SAMEA5159865
PRJEB18113	SAMEA5159863
PRJEB18113	SAMEA5159866
PRJEB18113	SAMEA4644735
PRJEB18113	SAMEA5159864
PRJEB18113	SAMEA19846918
PRJEB18113	SAMEA33668668
PRJEB18113	SAMEA5159835
PRJEB18113	SAMEA32999668
PRJEB18113	SAMEA32998168
PRJEB18113	SAMEA32989918
PRJEB18113	SAMEA4560538
PRJEB18113	SAMEA19847668
PRJEB18113	SAMEA19848418
PRJEB18113	SAMEA19849168
PRJEB18113	SAMEA5714967
PRJEB18113	SAMEA6528879
PRJEB28191	SAMEA6272106

PRJEB28191	SAMEA6272107
PRJEB28191	SAMEA6272109
PRJEB28191	SAMEA6272110
PRJEB28191	SAMEA6272111
PRJEB28191	SAMEA6272112
PRJEB28191	SAMEA6272113
PRJEB28191	SAMEA6272114
PRJEB28191	SAMEA6272115
PRJEB28191	SAMEA6272116
PRJEB28191	SAMEA6272118
PRJEB28191	SAMEA6272119
PRJEB28191	SAMEA6272120
PRJEB28191	SAMEA6272121
PRJEB28191	SAMEA6272122
PRJEB28191	SAMEA6272123
PRJEB28191	SAMEA6272124
PRJEB28191	SAMEA6272125
PRJEB28191	SAMEA6272126
PRJEB28191	SAMEA6272127
PRJEB28191	SAMEA6163175
PRJEB28191	SAMEA6163176
PRJEB28191	SAMEA6163177
PRJEB28191	SAMEA6163178
PRJEB28191	SAMEA6163179
PRJEB28191	SAMEA6163180
PRJEB28191	SAMEA6163181
PRJEB28191	SAMEA6163182
PRJEB28191	SAMEA6163183
PRJEB28191	SAMEA6163184
PRJEB28191	SAMEA6163186
PRJEB28191	SAMEA6163187
PRJEB28191	SAMEA6163188
PRJEB28191	SAMEA6163189
PRJEB28191	SAMEA6163190
PRJEB28191	SAMEA6163191
PRJEB28191	SAMEA6163192
PRJEB28191	SAMEA6163193
PRJEB28191	SAMEA6163194
PRJEB28191	SAMEA6163195
PRJEB28191	SAMEA6272082
PRJEB28191	SAMEA6272083
PRJEB28191	SAMEA6272084
PRJEB28191	SAMEA6272085

PRJEB28191	SAMEA6272086
PRJEB28191	SAMEA6272087
PRJEB28191	SAMEA6272088
PRJEB28191	SAMEA6272089
PRJEB28191	SAMEA6272090
PRJEB28191	SAMEA6272091
PRJEB28191	SAMEA6272092
PRJEB28191	SAMEA6272093
PRJEB28191	SAMEA6272094
PRJEB28191	SAMEA6272095
PRJEB28191	SAMEA6272096
PRJEB28191	SAMEA6272097
PRJEB28191	SAMEA6272098
PRJEB28191	SAMEA6272099
PRJEB28191	SAMEA6272100
PRJEB28191	SAMEA6272101
PRJEB28191	SAMEA6272102
PRJEB28191	SAMEA6272103
PRJEB28191	SAMEA6272104
PRJEB28191	SAMEA6272105
PRJEB18113	SAMEA5714968
PRJEB18113	SAMEA5714969
PRJEB14604	SAMEA4051548
PRJEB18113	SAMEA19849918
PRJEB18113	SAMEA5159890
PRJEB11963	SAMEA3682654
PRJEB18113	SAMEA4644737
PRJEB18113	SAMEA4644729
PRJEB18113	SAMEA4644732
PRJEB18113	SAMEA4644731
PRJEB18113	SAMEA19325668
PRJEB18113	SAMEA5159854
PRJEB18113	SAMEA6528904
PRJEB18113	SAMEA6528896
PRJEB18113	SAMEA4644746
PRJEB18113	SAMEA4644736
PRJEB18113	SAMEA5415500
PRJEB18113	SAMEA4560543
PRJEB18113	SAMEA5159879
PRJEB18113	SAMEA4644753
PRJEB11963	SAMEA3682653
PRJEB18113	SAMEA5415483
PRJEB18113	SAMEA5415484

PRJEB18113	SAMEA4644738
PRJEB18113	SAMEA5159857
PRJEB18113	SAMEA4644748
PRJEB18113	SAMEA5159855
PRJEB18113	SAMEA19309918
PRJEB18113	SAMEA5415503
PRJEB18113	SAMEA5159859
PRJEB18113	SAMEA5159860
PRJEB18113	SAMEA4644733
PRJEB18113	SAMEA4644760
PRJEB18113	SAMEA5159840
PRJEB18113	SAMEA5415501
PRJEB18113	SAMEA5415502
PRJEB18113	SAMEA19310668
PRJEB18113	SAMEA6528905
PRJEB18113	SAMEA4644761
PRJEB18113	SAMEA5714977
PRJEB18113	SAMEA5714978
PRJEB18113	SAMEA19322668
PRJEB18113	SAMEA6528907
PRJEB18113	SAMEA6528908
PRJEB18113	SAMEA6528909
PRJEB18113	SAMEA33004168
PRJEB18113	SAMEA5159878
PRJEB18113	SAMEA5159884
PRJEB18113	SAMEA19318168
PRJEB18113	SAMEA6528906
PRJEB18113	SAMEA19317418
PRJEB18113	SAMEA5159842
PRJEB18113	SAMEA33000418
PRJEB18113	SAMEA5160152
PRJEB18113	SAMEA5159839
PRJEB18113	SAMEA5159841
PRJEB18113	SAMEA5159844
PRJEB18113	SAMEA6528899
PRJEB18113	SAMEA19311418
PRJEB18113	SAMEA19316668
PRJEB18113	SAMEA19320418
PRJEB18113	SAMEA19321168
PRJEB18113	SAMEA19321918
PRJEB18113	SAMEA4644759
PRJEB18113	SAMEA4644751
PRJEB18113	SAMEA19312168

PRJEB18113	SAMEA5415492
PRJEB18113	SAMEA5415494
PRJEB18113	SAMEA5415496
PRJEB18113	SAMEA6528897
PRJEB18113	SAMEA6528902
PRJEB18113	SAMEA6528903
PRJEB18113	SAMEA5159880
PRJEB18113	SAMEA5159833
PRJEB18113	SAMEA5159882
PRJEB18113	SAMEA5159881
PRJEB18113	SAMEA5159883
	SAMN08612501
	SAMN08612529
	SAMN08612533
	SAMN08612544
PRJEB18113	SAMEA5159763
	SAMEA3390167
	SAMEA3390170
PRJEB18113	SAMEA32980918
PRJEB18113	SAMEA32981668
PRJEB18113	SAMEA32982418
	SAMEA3390189
	SAMEA3390191
PRJEB18113	SAMEA33001168
PRJEB5965	SAMEA2422242
PRJEB18113	SAMEA5159836
PRJEB18113	SAMEA19324918
PRJEB18113	SAMEA33001918
PRJEB18113	SAMEA5159845
PRJEB18113	SAMEA19852168
PRJEB18113	SAMEA19852918
PRJEB18113	SAMEA19853668
PRJEB12094	SAMEA3706829
PRJEB12094	SAMEA3706828
PRJEB18113	SAMEA4644767
PRJEB18113	SAMEA5160021
PRJEB18113	SAMEA4644726
PRJEB18113	SAMEA19864168
PRJEB18113	SAMEA19864918
PRJEB18113	SAMEA19865668
PRJEB18113	SAMEA19866418
PRJEB18113	SAMEA19867168
PRJEB18113	SAMEA19867918

PRJEB18113	SAMEA19868668
PRJEB18113	SAMEA19869418
PRJEB18113	SAMEA19870168
PRJEB18113	SAMEA19871668
PRJEB8226	SAMEA3209361
PRJEB7707	SAMEA3113485
PRJEB18113	SAMEA19874668
PRJEB18113	SAMEA32983168
PRJEB18113	SAMEA32983918
PRJEB18113	SAMEA32984668
PRJEB18113	SAMEA33669418
PRJEB18113	SAMEA19875418
PRJEB18113	SAMEA32985418
PRJEB18113	SAMEA19877668
PRJEB18113	SAMEA32988418
PRJEB18113	SAMEA32990668
PRJEB18113	SAMEA32992918
PRJEB18113	SAMEA32986918
PRJEB18113	SAMEA32991418
PRJEB18113	SAMEA32992168
PRJEB12092	SAMEA3706814
PRJEB12092	SAMEA3706815
PRJEB12092	SAMEA3706816
PRJEB18113	SAMEA32987668
PRJEB18113	SAMEA32989168
PRJEB18113	SAMEA32995168
PRJEB18113	SAMEA32995918
PRJEB18113	SAMEA32996668
PRJEB7528	SAMEA2821387
PRJEB7527	SAMEA2821386
PRJEB18113	SAMEA32997418
PRJEB12095	SAMEA3706830
PRJEB12094	SAMEA3706827
PRJEB18113	SAMEA6528884
PRJEB18113	SAMEA6528883
PRJEB18113	SAMEA6528882
PRJEB18113	SAMEA6528881
PRJEB18113	SAMEA6528885
	SAMN10598557
	SAMN10598558
PRJEB18113	SAMEA5714970
PRJEB18113	SAMEA5159831
PRJEB18113	SAMEA5159830

PRJEB18113	SAMEA5159829
PRJEB18113	SAMEA5564718
PRJEB12093	SAMEA3706826
PRJEB18113	SAMEA5564717
PRJEB18113	SAMEA5566449
PRJEB18113	SAMEA5564719
PRJEB18113	SAMEA5159856
PRJEB18113	SAMEA5564720
PRJEB18113	SAMEA5159762
PRJEB18113	SAMEA5159858
PRJEB18113	SAMEA5564722
PRJEB18113	SAMEA5564725
PRJEB18113	SAMEA5160153
PRJEB18113	SAMEA6528880
PRJEB18113	SAMEA4644747
PRJEB18113	SAMEA5564715
PRJEB18113	SAMEA5564713
PRJEB18113	SAMEA5564714
PRJEB18113	SAMEA5415499
PRJEB18113	SAMEA5159832
PRJEB18113	SAMEA5564711
PRJEB18113	SAMEA5564710
PRJEB18113	SAMEA5564724
PRJEB18113	SAMEA5564723
PRJEB18113	SAMEA5564721
PRJEB18113	SAMEA6528910
PRJEB18113	SAMEA4644744
PRJEB18113	SAMEA5564712
PRJEB18113	SAMEA4644745
PRJEB18113	SAMEA5159876
PRJEB18113	SAMEA5159877
PRJEB18113	SAMEA5415487
PRJEB18113	SAMEA33004918
PRJEB18113	SAMEA19309168
PRJEB18113	SAMEA19876168
PRJEB18113	SAMEA19876918
PRJEB11962	SAMEA3682652
PRJEB18113	SAMEA5159889
PRJEB18113	SAMEA5716181
PRJEB18113	SAMEA5564737
PRJEB18113	SAMEA5159888
PRJEB18113	SAMEA5564730
PRJEB18113	SAMEA5714980

PRJEB18113	SAMEA5564731
PRJEB18113	SAMEA5564729
PRJEB18113	SAMEA5564735
PRJEB18113	SAMEA5159887
PRJEB18113	SAMEA5564734
PRJEB18113	SAMEA5564732
PRJEB18113	SAMEA5564733
PRJEB18113	SAMEA5714981
PRJEB18113	SAMEA5564736
PRJEB5435	SAMEA2357050
PRJEB18113	SAMEA5159846
	SAMN08473804
	SAMN09379712
	SAMN09510423
PRJEB18113	SAMEA6528901
PRJEB18113	SAMEA5415493
PRJEB18113	SAMEA5415495
PRJEB18113	SAMEA5415497
PRJEB18113	SAMEA5714973
PRJEB12093	SAMEA3706824
PRJEB18113	SAMEA19872418
PRJEB18113	SAMEA5159851
PRJEB18113	SAMEA6528898
PRJEB18113	SAMEA6528900
