# Supplementary material for: A de novo mutation in KRT5 in a crossbred calf with epidermolysis bullosa simplex
Source: J Vet Intern Med. 2020 Nov 2;34(6):2800–7. doi: 10.1111/jvim.15943 (PMC7694802; doi:10.1111/jvim.15943)
Supplement: Supplementary file 3 — Table S3 List of the remaining variants after the comparison to the global control cohort of 3103 genomes of other breeds (1000 Bull Genomes Project run 7; www.1000bullgenomes.com), revealing 26 protein‐changing variants only present in the genome of the EBS‐affected calf. These 26 variants with a moderate or high predicted impact on the encoded protein were located within 25 different genes or loci. Note that the predicted pathogenic variant NM_001008663.1:c.534_536delCAA is the only 1 located in a candidate gene. [file JVIM-34-2800-s003.pdf]

#CHROM	POS	REF	ALT	GENOTYPE	EFFECT	IMPACT	GENE	GENEID	FEATUREID	EXON	CDNA	PROTEIN
11	119764	C	T	0/1	missense_variant	MODERATE	*ZC3H6*	506237	XM_002707712.5	12	c.3307G>A	p.Gly1103Ser
11	119764	C	T	0/1	missense_variant	MODERATE	*ZC3H6*	506237	XM_024999265.1	10	c.2920G>A	p.Gly974Ser
1	144009178	G	A	0/1	missense_variant	MODERATE	*AGPAT3*	506607	NM_001038046.1	3	c.121G>A	p.Val41Met
11	67182753	G	C	0/1	missense_variant	MODERATE	*ARHGAP25*	534994	NM_001046392.1	11	c.1846G>C	p.Glu616Gln
11	67182753	G	C	0/1	missense_variant	MODERATE	*ARHGAP25*	534994	XM_005212861.4	11	c.1855G>C	p.Glu619Gln
11	67182753	G	C	0/1	missense_variant	MODERATE	*ARHGAP25*	534994	XM_010810125.3	10	c.1738G>C	p.Glu580Gln
11	67182753	G	C	0/1	missense_variant	MODERATE	*ARHGAP25*	534994	XM_010810126.3	8	c.1492G>C	p.Glu498Gln
11	67182753	G	C	0/1	missense_variant	MODERATE	*ARHGAP25*	534994	XM_015473507.2	11	c.1864G>C	p.Glu622Gln
11	67182753	G	C	0/1	missense_variant	MODERATE	*ARHGAP25*	534994	XM_024998860.1	10	c.1588G>C	p.Glu530Gln
11	70586568	G	A	0/1	missense_variant	MODERATE	*ALK*	536642	XM_024999478.1	11	c.1936G>A	p.Gly646Arg
12	71214560	G	A	0/1	missense_variant	MODERATE	*LOC522174*	522174	XM_025000204.1	6	c.707C>T	p.Ser236Phe
13	2377500	G	A	0/1	missense_variant	MODERATE	*PLCB4*	281985	XM_025000316.1	12	c.731G>A	p.Ser244Asn
13	2377500	G	A	0/1	missense_variant	MODERATE	*PLCB4*	281985	XM_025000317.1	10	c.695G>A	p.Ser232Asn
13	2377500	G	A	0/1	missense_variant	MODERATE	*PLCB4*	281985	XM_025000319.1	11	c.749G>A	p.Ser250Asn
13	2377500	G	A	0/1	missense_variant	MODERATE	*PLCB4*	281985	XM_025000320.1	6	c.302G>A	p.Ser101Asn
13	65570602	T	G	0/1	splice_donor_variant&intron_variant	HIGH	*DLGAP4*	520521	XM_010811464.2	1	c.85+2T>G	
15	28053129	T	C	0/1	missense_variant&splice_region_variant	MODERATE	*CEP164*	514284	XM_015474629.2	13	c.1397T>C	p.Leu466Ser
15	28053129	T	C	0/1	missense_variant&splice_region_variant	MODERATE	*CEP164*	514284	XM_024975763.1	11	c.1241T>C	p.Leu414Ser
15	28053129	T	C	0/1	missense_variant&splice_region_variant	MODERATE	*CEP164*	514284	XM_024975764.1	11	c.1232T>C	p.Leu411Ser
15	28053129	T	C	0/1	missense_variant&splice_region_variant	MODERATE	*CEP164*	514284	XM_024975765.1	8	c.851T>C	p.Leu284Ser
15	28053129	T	C	0/1	missense_variant&splice_region_variant	MODERATE	*CEP164*	514284	XM_024975767.1	8	c.842T>C	p.Leu281Ser
16	35067966	C	G	0/1	missense_variant&splice_region_variant	MODERATE	*WDR64*	783230	NM_001205438.2	11	c.1292G>C	p.Gly431Ala
16	35067966	C	G	0/1	missense_variant&splice_region_variant	MODERATE	*WDR64*	783230	XM_024976544.1	12	c.1322G>C	p.Gly441Ala
16	62172664	C	G	0/1	missense_variant	MODERATE	*MR1*	506206	NM_001190298.1	2	c.153C>G	p.Ile51Met
25	2663294	GCA	G	0/1	frameshift_variant	HIGH	*LOC539468*	539468	XM_015460305.2	2	c.644_645delCA	p.Ala215fs
25	2663297	G	GTT	0/1	frameshift_variant	HIGH	*LOC539468*	539468	XM_015460305.2	2	c.646_647insTT	p.Ala216fs
29	44872199	C	A	0/1	missense_variant	MODERATE	*LRFN4*	530084	XM_005227110.4	2	c.853C>A	p.Arg285Ser
29	48694869	C	T	0/1	missense_variant	MODERATE	*SLC22A18*	504885	XM_005227308.4	10	c.1009G>A	p.Gly337Ser
29	48694869	C	T	0/1	missense_variant	MODERATE	*SLC22A18*	504885	XM_024987010.1	8	c.1012G>A	p.Gly338Ser
3	100074635	C	T	0/1	missense_variant	MODERATE	*MAST2*	535643	XM_003585907.4	25	c.3326G>A	p.Arg1109Gln
3	100074635	C	T	0/1	missense_variant	MODERATE	*MAST2*	535643	XM_005204880.3	26	c.3347G>A	p.Arg1116Gln
3	100074635	C	T	0/1	missense_variant	MODERATE	*MAST2*	535643	XM_005204883.4	24	c.3071G>A	p.Arg1024Gln
3	100074635	C	T	0/1	missense_variant	MODERATE	*MAST2*	535643	XM_005204884.4	23	c.3050G>A	p.Arg1017Gln
3	100074635	C	T	0/1	missense_variant	MODERATE	*MAST2*	535643	XM_005204886.4	23	c.2876G>A	p.Arg959Gln
3	100074635	C	T	0/1	missense_variant	MODERATE	*MAST2*	535643	XM_010803741.3	24	c.2897G>A	p.Arg966Gln

3	100074635	C	T	0/1	missense_variant	MODERATE	*MAST2*	535643	XM_024990152.1	26	c.3164G>A	p.Arg1055Gln
3	54768870	C	T	0/1	missense_variant	MODERATE	*LOC507055*	507055	XM_002686269.6	5	c.541C>T	p.Arg181Trp
3	54768870	C	T	0/1	missense_variant	MODERATE	*LOC507055*	507055	XM_015464659.2	4	c.304C>T	p.Arg102Trp
4	93400822	C	T	0/1	missense_variant	MODERATE	*SMKR1*	784270	XM_024991157.1	3	c.113C>T	p.Ala38Val
5	115804850	G	C	0/1	missense_variant	MODERATE	*SMC1B*	522122	XM_002687980.4	5	c.622C>G	p.Arg208Gly
5	14697177	T	C	0/1	missense_variant	MODERATE	*LRRIQ1*	781474	XM_005206079.4	8	c.2213T>C	p.Ile738Thr
5	27367604	TCAA	T	0/1	disruptive_inframe_deletion	MODERATE	*KRT5*	281268	NM_001008663.1	1	c.534_536delCAA	p.Asn178del
5	27898486	G	A	0/1	missense_variant	MODERATE	*ACVR1B*	539315	XM_024992431.1	2	c.104C>T	p.Ala35Val
5	29727679	T	C	0/1	missense_variant	MODERATE	*LIMA1*	540637	NM_001192754.1	11	c.2108T>C	p.Leu703Pro
5	29727679	T	C	0/1	missense_variant	MODERATE	*LIMA1*	540637	XM_005206324.4	11	c.2105T>C	p.Leu702Pro
5	29727679	T	C	0/1	missense_variant	MODERATE	*LIMA1*	540637	XM_005206325.4	11	c.2102T>C	p.Leu701Pro
5	29727679	T	C	0/1	missense_variant	MODERATE	*LIMA1*	540637	XM_024992054.1	9	c.1241T>C	p.Leu414Pro
5	83875046	C	T	0/1	missense_variant	MODERATE	*RASSF8*	515639	XM_024991718.1	4	c.817G>A	p.Glu273Lys
7	27372306	G	A	0/1	stop_gained	HIGH	*GRAMD2B*	505627	NM_001079579.1	9	c.844C>T	p.Arg282*
7	27372306	G	A	0/1	stop_gained	HIGH	*GRAMD2B*	505627	XM_005209088.4	9	c.889C>T	p.Arg297*
7	5889843	C	A	0/1	splice_donor_variant&intron_variant	HIGH	*MYO9B*	513493	NM_001193096.2	33	c.5254+1G>T	
7	5889843	C	A	0/1	splice_donor_variant&intron_variant	HIGH	*MYO9B*	513493	XM_005208501.4	34	c.5347+1G>T	
7	5889843	C	A	0/1	splice_donor_variant&intron_variant	HIGH	*MYO9B*	513493	XM_010806569.3	34	c.5344+1G>T	
7	5889843	C	A	0/1	splice_donor_variant&intron_variant	HIGH	*MYO9B*	513493	XM_010806571.3	34	c.5314+1G>T	
7	5889843	C	A	0/1	splice_donor_variant&intron_variant	HIGH	*MYO9B*	513493	XM_010806573.3	33	c.5257+1G>T	
7	5889843	C	A	0/1	splice_donor_variant&intron_variant	HIGH	*MYO9B*	513493	XM_010806575.3	33	c.5233+1G>T	
7	5889843	C	A	0/1	splice_donor_variant&intron_variant	HIGH	*MYO9B*	513493	XM_024994174.1	33	c.5251+1G>T	
9	88398528	C	T	0/1	missense_variant	MODERATE	*AKAP12*	513774	XM_010808687.3	2	c.3611C>T	p.Pro1204Leu
9	88398528	C	T	0/1	missense_variant	MODERATE	*AKAP12*	513774	XM_024997062.1	4	c.3860C>T	p.Pro1287Leu
9	88398528	C	T	0/1	missense_variant	MODERATE	*AKAP12*	513774	XM_024997063.1	2	c.3608C>T	p.Pro1203Leu
